# Carbapenem-resistant *Acinetobacter baumannii* bloodstream infections in critically ill patients: prognostic factors and development of a nomogram

**DOI:** 10.3389/fmed.2026.1781326

**Published:** 2026-02-25

**Authors:** Lihua Huang, Wei Gu, Sanhu Wang, Fuxing Li

**Affiliations:** 1 Department of Infectious Diseases, The First Affiliated Hospital of Dali University, Dali, Yunnan, China; 2 Department of Jiangxi Provincial Key Laboratory of Medicine, Clinical Laboratory of the Second Affiliated Hospital of Nanchang University, Nanchang, Jiangxi, China; 3 Department of Clinical Laboratory, Hunan University of Medicine General Hospital, Huaihua, Hunan, China

**Keywords:** 30-day mortality, bloodstream infection, carbapenem-resistant *Acinetobacter baumannii*, intensive care unit, nomogram, risk factors

## Abstract

**Objective:**

This retrospective study aimed to identify the clinical features and prognostic determinants in intensive care unit (ICU) patients with carbapenem-resistant *Acinetobacter baumannii* (CRAB) bloodstream infection (BSI) and to establish a personalized risk prediction model.

**Methods:**

This retrospective cohort study included 185 ICU patients with CRAB-BSI at a tertiary care hospital between 2013 and 2023. Based on 30-day outcomes, patients were categorized into survival and non-survival groups. Independent risk factors for mortality were identified through univariate and multivariate logistic regression analyses. These factors were used to construct a nomogram prediction model. Model performance was evaluated by assessing discrimination using the area under the receiver operating characteristic curve with a calibration plot, and clinical utility via decision curve analysis.

**Results:**

The 30-day mortality rate among ICU patients with CRAB-BSI was 60.0%. Multivariate analysis revealed that age [odds ratio (OR) = 1.04, 95% confidence interval (CI): 1.02–1.07], impaired consciousness (OR = 5.10, 95% CI: 2.27–11.45), prior corticosteroid use (OR = 5.82, 95% CI: 2.10–16.12), Sequential Organ Failure Assessment (SOFA) score (OR = 1.26, 95% CI: 1.12–1.42), and C-reactive protein (CRP) level (OR = 1.01, 95% CI: 1.01–1.02) were independent risk factors for 30-day mortality. A nomogram incorporating these variables achieved an area under the curve (AUROC) of 0.863 for predicting 30-day mortality risk. The calibration curve indicated excellent concordance between predictions and observed outcomes, and decision curve analysis demonstrated significant clinical net benefit over a wide range of probability thresholds.

**Conclusion:**

Mortality is high in ICU patients with CRAB-BSI and is significantly associated with age, impaired consciousness, corticosteroid use, SOFA score, and CRP level. The developed nomogram exhibits strong predictive accuracy and may function as a practical tool for quantitative management decisions.

## Introduction

*Acinetobacter baumannii* is a significant Gram-negative opportunistic pathogen in healthcare-associated infections, notorious for its robust environmental persistence and remarkable potential to acquire antimicrobial resistance (1–4). Unlike other *Acinetobacter* species that often act as environmental or skin commensals, clinically isolated *A. baumannii* is unequivocally pathogenic, predominantly infecting hospitalized critically ill patients and posing a severe threat, particularly within intensive care units (ICUs) (3, 5–7). Its ability to persist on inanimate surfaces and form biofilms not only facilitates enduring presence and spread in hospital settings but also frequently leads to diverse serious infections, including ventilator-associated pneumonia (VAP) (8–10), bloodstream infections (BSI) (3, 11, 12), wound infections, and meningitis (13–15). Notably, pneumonia represents the most common clinical manifestation of *Acinetobacter* infection. Globally, the majority of *A. baumannii* strains causing hospital-acquired pneumonia/ventilator-associated pneumonia (HAP/VAP) are multidrug-resistant, including carbapenem-resistant *A. baumannii* (CRAB), with a recent meta-analysis reporting a pooled prevalence of 79.9% (95% CI 73.9–85.4%) (16). Of greater concern is the propensity of *A. baumannii* to develop multidrug resistance. While carbapenems were historically considered last-line therapeutic agents for its infections (17), current global surveillance data indicate alarmingly high levels of carbapenem resistance. Consequently, the World Health Organization has designated CRAB a “critical priority” pathogen, leaving clinicians with severely limited therapeutic options (2, 3, 18).

This therapeutic impasse directly translates into dire clinical outcomes. Carbapenem-resistant *A. baumannii* bloodstream infection (CRAB-BSI) is associated with exceedingly high mortality. Surveillance data show that the global prevalence of CRAB approaches 45%, with infection rates exceeding 70% among clinical isolates in China (3, 19). Once CRAB-BSI develops, mortality in ICU patients can surpass 60%, a risk significantly higher than that associated with bloodstream infections caused by other multidrug-resistant Gram-negative bacteria (19–21). It is noteworthy that compared to other pathogens causing ICU-acquired BSIs, *A. baumannii*, although less frequent, is most often healthcare-associated and is the pathogen most likely to exhibit carbapenem resistance (22). This elevated mortality stems from the convergence of two principal factors. On one hand, the scarcity of effective antimicrobial agents makes initial empirical treatment failure or delay common (1, 23, 24). On the other hand, these infections almost exclusively occur in critically ill patients with severe underlying conditions, organ dysfunction, or immunosuppression, whose physiological reserve is already profoundly depleted (25–27). Furthermore, patient-to-patient transmission serves as a primary driver of colonization and infection, particularly during outbreaks (28).

The COVID-19 pandemic may have further exacerbated this crisis (29–31). A significant surge in CRAB infections within ICUs was observed globally during the pandemic. For instance, in Europe, bloodstream infections caused by *Acinetobacter* spp. increased by 57%, and their carbapenem resistance rate rose from 48.4% in 2018–2019 to 65.8% in 2020–2021 (18, 32). The pandemic led to a massive influx of patients with viral pneumonia into ICUs, where widespread glucocorticoid use and prolonged hospitalization, coupled with the strain on infection control measures due to system overload, have been associated with increased CRAB infection rates in multiple studies (33–35). Therefore, in managing CRAB-BSI—characterized by limited treatment options and complex host factors—a pivotal preliminary step in clinical decision-making is the accurate early identification of patients at the highest risk of mortality. Early and precise risk stratification is becoming increasingly crucial for guiding decisions on initiating intensive treatment regimens (e.g., combination therapy or novel agents) and for the rational allocation of critical care resources.

Despite the clear importance of accurate risk assessment, practical tools readily applicable at the ICU bedside remain scarce. While recent studies have explored complex algorithms, including machine learning, to predict outcomes in CRAB infections, the integration of such models into time-pressured clinical workflows is often hindered by their “black-box” nature, reliance on extensive specific variables, and computational complexity (36, 37). Consequently, within the realm of CRAB-BSI management, there is a pressing need for a tool that leverages routinely available clinical parameters, is intuitive, and can rapidly provide an individualized risk score to inform early clinical decisions. To address this gap, this study aims to identify independent risk factors for 30-day mortality following CRAB-BSI in ICU patients through a retrospective cohort analysis and to subsequently develop and validate a visual nomogram prediction model. The goal is to provide clinicians with a concise and practical tool for risk assessment.

## Materials and methods

### Study subjects

**Figure 1:**
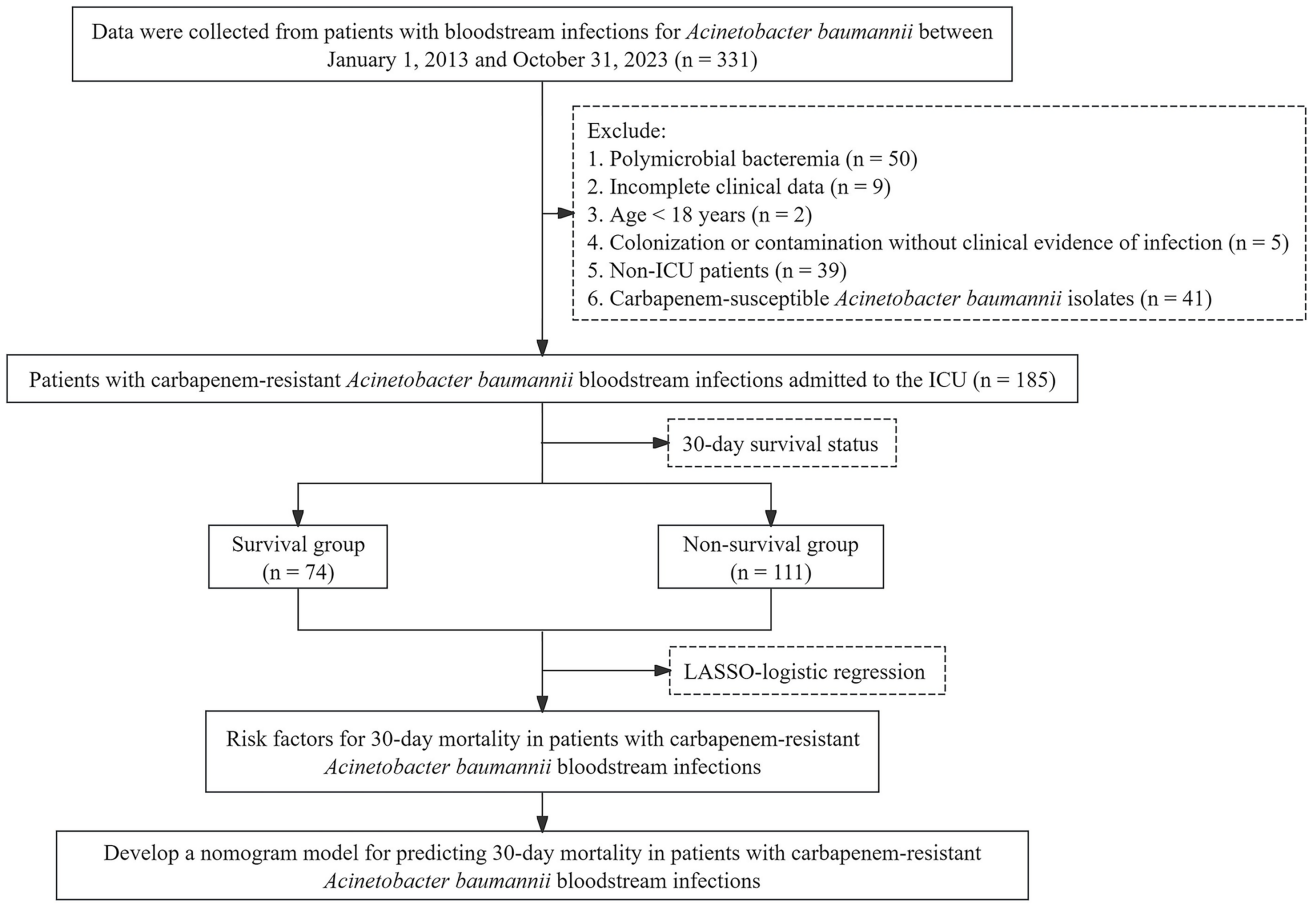
Participant flow diagram.

This retrospective cohort study included all patients diagnosed with CRAB-BSI in the ICU of the First Affiliated Hospital of Dali University between January 1, 2013, and October 31, 2023. The inclusion criteria were as follows: (1) Blood culture yielding a single pathogen, identified as carbapenem-resistant *Acinetobacter baumannii* (CRAB) demonstrating resistance to imipenem or meropenem, blood culture positivity on at least one occasion, accompanied by clinical signs of bloodstream infection (BSI); for patients with multiple positive cultures, only the first isolate of CRAB was included. (2) Fulfillment of the clinical diagnostic criteria for CRAB-BSI based on clinical symptoms, confirmed by laboratory tests, and availability of complete clinical data for analysis and prognosis assessment. The exclusion criteria are presented in Figure 1. The included patients were classified based on their 30-day outcomes.

### Data collection

Data were retrospectively extracted from the Electronic Medical Record system and compiled into a structured dataset using Microsoft Excel (Excel for MacOS, 2023). Collected variables encompassed demographic characteristics, clinical signs and laboratory indicators (recorded on the day of positive Blood culture), neurological status (such as impaired consciousness), primary diagnosis and comorbidities [assessed using the age-adjusted Charlson Comorbidity Index (aCCI)], and details of medical interventions and pharmacological treatments. A history of glucocorticoid use was defined as any documented administration of systemic corticosteroids (e.g., prednisone, methylprednisolone, hydrocortisone) via a non-topical route (oral, intravenous, or intramuscular), recorded in the medical history prior to or initiated during the current hospitalization but before the onset of CRAB-BSI. For the purpose of this study, this binary variable aimed to capture any potential exposure to iatrogenic immunosuppression; no minimum dose or duration threshold was applied to ensure sensitivity in this retrospective design. Disease severity was quantified using the Pitt bacteremia score and the Sequential Organ Failure Assessment (SOFA) score, both assessed on the day of bacteremia onset. Additional data included the total length of hospitalization and the primary outcome of 30-day mortality. All data were de-identified prior to the analysis to ensure patient confidentiality.

### Bacterial identification and antimicrobial susceptibility testing

*Acinetobacter baumannii* isolates were identified using matrix-assisted laser desorption/ionization time-of-flight mass spectrometry (MALDI-TOF MS; Vitek MS, BioMérieux, France) or the Vitek 2 automated system (BioMérieux, France) in accordance with the manufacturer’s instructions. Antimicrobial susceptibility testing (AST) was conducted using the ATB system (BioMérieux, France) or the Kirby-Bauer disk diffusion method. The minimum inhibitory concentration was determined via the broth microdilution method. All AST procedures and result interpretations were performed in accordance with the Clinical and Laboratory Standards Institute (CLSI) guidelines (M100 series) applicable during the respective study years. *Pseudomonas aeruginosa* ATCC 27853 served as the quality control strain to ensure the accuracy of the AST procedures.

### Statistical analysis

All statistical analyses were performed using the Statistical Package for the Social Sciences software (version 26.0). Patients with missing data for key analytical variables were excluded, resulting in a complete-case dataset for all subsequent analyses. The normality of continuous variables was assessed using the *Shapiro*–*Wilk* test. Normally distributed data are presented as mean ± standard deviation, non-normally distributed data as median (interquartile range), and categorical variables as frequency (percentage). Intergroup comparisons were performed using the independent samples t-test for normally distributed continuous variables, the *Mann*–*Whitney U* test for non-normally distributed continuous variables, and the *chi-square* test for categorical variables. To identify factors associated with 30-day outcomes in patients with CRAB-BSI, least absolute shrinkage and selection operator (LASSO) regression was first employed for variable screening. The variables retained in the LASSO analysis were subsequently entered into a multivariate binary logistic regression model to determine the independent risk factors for 30-day mortality. A nomogram prediction model was constructed based on these risk factors. For internal validation of the nomogram and to correct for potential overfitting, a bootstrap resampling procedure with 1,000 replicates was performed. This method was used to assess the stability and generalizability of the model performance metrics. Model discrimination was evaluated using the area under the receiver operating characteristic curve (AUROC), and the consistency index (C-index), and sensitivity and specificity were also calculated. All statistical tests were two-sided, and a *p* < 0.05 was considered statistically significant.

## Results

### Baseline characteristics and grouping of CRAB-BSI patients

A total of 331 patients with *Acinetobacter baumannii* BSI were initially screened. After applying the inclusion and exclusion criteria (Figure 1), 185 ICU patients with CRAB-BSI were included in the final analysis. Based on 30-day outcomes, 74 (40.0%) patients were classified as survivors and 111 (60.0%) as non-survivors. Comparative analysis of baseline characteristics (Table 1) revealed that the non-survival group had a significantly higher mean age than the survival group (64.21 ± 13.48 years versus 54.55 ± 15.91 years). The non-survival group also had a higher proportion of patients with impaired consciousness (58.56% versus 22.97%), a greater history of carbapenem exposure (58.56% versus 9.46%), higher median SOFA scores [6.00 (3.00–9.00) versus 4.00 (1.00–7.00)], and a notably shorter median hospital stay [17.00 (9.00–30.00) days versus 29.00 (20.00–47.00) days]. All these differences were statistically significant (all *p* < 0.05).

**Table 1:** Patient demographics and baseline characteristics in 185 patients with CRAB-BSI admitted to the ICU.

Parameter	Survival (*n* = 74)	Non-survival (*n* = 111)	χ^2^/t/z	*p-*value
Patient characteristics				
Age (years), Mean ± SD	54.55 ± 15.91	64.21 ± 13.48	−4.44	< 0.001
Sex (male), *n* (%)	57.00 (77.03%)	75.00 (67.57%)	1.94	0.248
Constants				
Body temperature (°C)	38.60 (38.00, 39.00)	38.70 (38.00, 39.00)	−0.76	0.446
Pulse rate (beats/min)	92.00 (78.25, 105.00)	96.00 (86.50, 109.50)	−1.30	0.194
Systolic blood pressure (mmHg)	115.34 ± 15.75	110.03 ± 25.77	1.74	0.084
Diastolic blood pressure (mmHg)	68.00 (60.25, 73.50)	65.00 (56.00, 72.50)	−1.43	0.152
Disturbance of consciousness, *n* (%)	17 (22.97%)	65 (58.56%)	22.78	< 0.001
Predominant underlying condition, *n* (%)				
Diabetes mellitus	10 (13.51%)	21 (18.92%)	0.93	0.335
Tumor	10 (13.51%)	13 (11.71%)	0.13	0.716
Hypertension	26 (35.14%)	37 (33.33%)	0.06	0.800
Coronary artery disease	5 (6.76%)	3 (2.70%)	0.92	0.337
Medical interventions, *n* (%)				
Radiotherapy and/or chemotherapy	3 (4.05%)	5 (4.50%)	0.00	> 0.999
Indwelling gastric tube	27 (36.49%)	44 (39.64%)	0.19	0.666
Indwelling urinary catheter	30 (40.54%)	50 (45.05%)	0.37	0.545
Central venous catheterization	17 (22.97%)	24 (21.62%)	0.05	0.828
CRRT	14 (18.92%)	29 (26.13%)	1.29	0.256
Mechanical ventilation	47 (63.51%)	80 (72.07%)	1.51	0.219
Surgery	27 (36.49%)	38 (34.23%)	0.10	0.753
Pharmacological therapy, *n* (%)				
Use glucocorticoids	7 (9.46%)	65 (58.56%)	45.03	< 0.001
History of carbapenem use	52 (70.27%)	95 (85.59%)	6.38	0.012
Combination antibiotic therapy	69 (93.24%)	107 (96.40%)	6.38	0.530
Disease severity, M (Q1, Q3)				
Pitt score	1.00 (0.00, 3.00)	1.00 (1.00, 3.00)	−1.07	0.284
SOFA score	4.00 (1.00, 7.00)	6.00 (3.00, 9.00)	−4.19	< 0.001
aCCI score	4.00 (2.00, 5.75)	4.00 (2.00, 5.00)	−0.93	0.355
ICU stays [days, M (Q1, Q3)]	11.00 (0.00, 22.00)	12.00 (6.00, 20.00)	−0.82	0.414
Hospital stays [days, M (Q1, Q3)]	29.00 (20.00, 47.00)	17.00 (9.00, 30.00)	−4.95	< 0.001

CRRT: Continuous renal replacement therapy; Pitt score, Pitt bacteremia score; SOFA, sequential organ failure assessment; aCCI, age-adjusted Charlson comorbidity index; ICU: Intensive Care Unit. Data are presented as mean ± standard deviation or median (interquartile range) as indicated; M, median.

### Laboratory parameters and antimicrobial susceptibility profiles

Laboratory findings on the day of infection onset are presented in Table 2. Compared to the survival group, the non-survival group had significantly higher median levels of C-reactive protein [119.00 (77.20–149.50) mg/L versus 85.18 (43.18–116.50) mg/L], procalcitonin [8.66 (2.78–17.54) ng/mL versus 3.06 (1.09–11.45) ng/mL], and creatinine [102.67 (69.70–163.17) μmol/L versus 82.53 (59.77–113.91) μmol/L]. Conversely, median platelet count [105.00 (71.50–170.00) × 10^9^/L versus 142.50 (101.25–186.75) × 10^9^/L] and median albumin level [30.58 (29.27–33.59) g/L versus 32.30 (30.79–35.60) g/L] were significantly lower.

**Table 2:** Laboratory indicators of patients in the 30-day survival group and the non-survival group with CRAB-BSI admitted to the ICU.

Parameter	Survival (*n* = 74)	Non-survival (*n* = 111)	*z*	*p*-value
C-reactive protein [mg/L, M (Q1, Q3)]	85.18 (43.18, 116.50)	119.00 (77.20, 149.50)	−3.80	< 0.001
Procalcitonin [ng/mL, M (Q1, Q3)]	3.06 (1.09, 11.45)	8.66 (2.78, 17.54)	−2.63	0.008
White blood cell [x 10^9^/L, M (Q1, Q3)]	12.58 (8.44, 17.54)	12.17 (7.06, 17.06)	−1.22	0.223
Hemoglobin [g/L, M (Q1, Q3)]	93.50 (78.25, 112.00)	90.00 (77.50, 108.50)	−0.74	0.461
Platelet [x 10^9^/L, M (Q1, Q3)]	142.50 (101.25, 186.75)	105.00 (71.50, 170.00)	−2.58	0.010
Neutrophil percentage [%, M (Q1, Q3)]	87.75 (82.25, 92.35)	85.50 (78.50, 90.80)	−1.91	0.056
Albumin [g/L, M (Q1, Q3)]	32.30 (30.79, 35.60)	30.58 (29.27, 33.59)	−2.80	0.005
Total bilirubin [μmol/L, M (Q1, Q3)]	15.82 (10.56, 24.97)	18.40 (12.12, 42.38)	−1.89	0.059
Creatinine [μmol/L, M (Q1, Q3)]	82.53 (59.77, 113.91)	102.67 (69.70,163.17)	−2.03	0.042

Data are presented as median (interquartile range); M, median.

The antimicrobial susceptibility testing (AST) results are presented in Table 3. Among the isolates tested, resistance rates were exceedingly high for most agents. For carbapenems, 97.8% (181/185) of isolates tested against imipenem were resistant, and 97.4% (37/38) of isolates tested against meropenem were resistant. Notably, the four isolates that were susceptible to imipenem were all confirmed to be resistant to meropenem, ensuring that all isolates included in the analysis exhibited resistance to at least one of these two key carbapenems. Resistance rates also exceeded 90% for most tested cephalosporins and quinolones. Only tigecycline, minocycline, polymyxin, and cefoperazone/sulbactam maintained relatively low resistance rates. No significant differences were observed in the resistance profiles between the survival and non-survival groups for any antibiotic.

**Table 3:** Antimicrobial susceptibility testing results (resistance rates) in 185 patients with CRAB-BSI admitted to the ICU.

Antimicrobial (*n*, %)	Total (*n* = 185)	Survival (*n* = 74)	Non-survival (*n* = 111)	*p-*value
Ampicillin (30 vs. 37)*	67 (100.00%)	30 (100.00%)	37 (100.00%)	> 0.999
Piperacillin (14 vs. 18)*	31 (96.88%)	14 (100%)	17 (94.44%)	> 0.999
Imipenem (74 vs. 111)*	181 (97.84%)	74 (100.00%)	107 (96.40%)	0.256
Meropenem (20 vs. 18)*	37 (97.37%)	20 (100.00%)	17 (94.44%)	0.957
Cefazolin (39 vs. 55)*	94 (100.00%)	39 (100.00%)	55 (100.00%)	> 0.999
Cefepime (73 vs. 110)*	177 (96.72%)	72 (98.63%)	105 (95.45%)	0.449
Cefoxitin (38 vs. 49)*	87 (100.00%)	38 (100.00%)	49 (100.00%)	> 0.999
Ceftriaxone (48 vs. 79)*	127 (100.00%)	48 (100.00%)	79 (100.00%)	> 0.999
Ceftazidime (48 vs. 57)*	103 (98.10%)	47 (97.92%)	56 (98.25%)	> 0.999
Cefotetan (19 vs. 23)*	42 (100.00%)	19 (100.00%)	23 (100.00%)	> 0.999
Cefotaxime (18 vs. 16)*	34 (100.00%)	18 (100.00%)	16 (100.00%)	> 0.999
Ciprofloxacin (64 vs. 88)*	149 (98.03%)	62 (96.88%)	87 (98.86%)	0.780
Levofloxacin (74 vs. 111)*	125 (67.57%)	54 (72.97%)	71 (63.96%)	0.699
Gentamicin (57 vs. 94)*	140 (92.72%)	52 (91.23%)	88 (93.62%)	0.822
Amikacin (12 vs. 17)*	26 (89.66%)	11 (91.67%)	15 (88.24%)	> 0.999
Tobramycin (65 vs. 94)*	138 (86.79%)	56 (86.15%)	82 (87.23%)	0.843
Tigecycline (48 vs. 79)*	12 (9.45%)	5 (10.42%)	7 (8.86%)	> 0.999
Minocycline (28 vs. 32)*	18 (30.00%)	8 (28.57%)	10 (31.25%)	0.821
Doxycycline (17 vs. 16)*	29 (87.88%)	16 (94.12%)	13 (81.25%)	0.550
Sulfamethoxazole (74 vs. 110)*	156 (84.78%)	64 (86.49%)	92 (83.64%)	0.598
Polymyxin (25 vs. 34)*	0 (0%)	0 (0%)	0 (0%)	> 0.999
Macrodantin (39 vs. 55)*	94 (100.00%)	39 (100.00%)	55 (100.00%)	> 0.999
Ampicillin/sulbactam (30 vs. 39)*	58 (84.06%)	27 (90.00%)	31 (79.49%)	0.395
Cefperazone/sulbactam (34 vs. 44)*	36 (46.15%)	17 (50.00%)	19 (43.18%)	0.549
Piperacillin/tazobactam (58 vs. 68)*	126 (99.21%)	58 (100.00%)	67 (98.53%)	> 0.999
Ticarcillin/clavulnic acid (20 vs. 17)*	37 (100.00%)	20 (100.00%)	17 (100.00%)	> 0.999
Amoxicillin/clavulnic acid (11 vs. 14)*	25 (100.00%)	11 (100.00%)	14 (100.00%)	> 0.999

*For each antibiotic, the numbers in parentheses (e.g., “Imipenem (74 vs 111)”) denote the number of CRAB isolates tested for susceptibility to that specific drug in the survival group versus the non-survival group, respectively. The values in the “Total,” “Survival,” and “Non-survival” columns represent the number (and percentage) of resistant isolates among those tested. Percentages are calculated as (number of resistant isolates/number of isolates tested) × 100% for the respective group or total.

### Analysis of risk factors for 30-day mortality in ICU patients with CRAB-BSI

**Figure 2:**
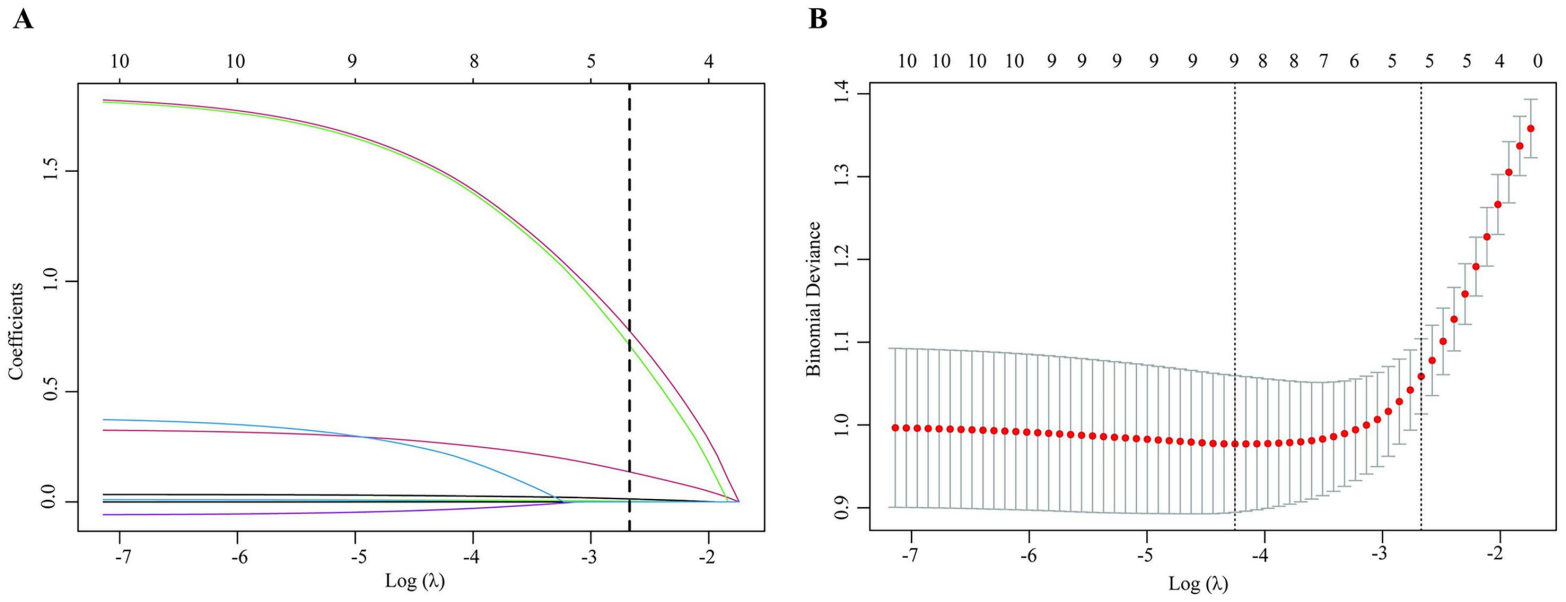
Selection of predictor variables using LASSO regression. **(A)** Coefficient profiles of the 10 candidate variables included in the LASSO regression. Each curve represents the trajectory of a variable’s coefficient as the penalty (*λ*) increases. The vertical dashed line is drawn at the value selected by the one-standard-error rule (λ.1se). **(B)** Ten-fold cross-validation curve for the LASSO regression. The left dashed vertical line indicates λ.min, the value of λ that gives the minimum mean cross-validated error. The right dashed vertical line indicates λ.1se, the largest value of λ such that the error is within one standard error of the minimum. This more parsimonious model (λ.1se), which selects fewer variables, was chosen for final analysis. In this study, at λ.1se (*λ* = 0.069), seven non-zero coefficient predictors were retained.

To identify independent risk factors, LASSO regression was first applied to the 10 variables that demonstrated significant differences in the univariate analysis for variable screening (Figure 2A). Using 10-fold cross-validation (optimal *λ* = 0.069), seven non-zero coefficient predictors were selected: age, impaired consciousness, history of carbapenem use, history of corticosteroid use, SOFA score, CRP, and ALB (Figure 2B). The univariate logistic regression results for these seven variables are detailed in Table 4.

**Table 4:** Univariate and multivariate logistic regression analysis of 30-day mortality risk factors in CRAB-BSI patients in the ICU.

Variables	Univariate logistic regression analysis					Multivariate logistic regression analysis				
	*β*	S.E	*Z*	*p*	OR (95%CI)	*β*	S.E	*Z*	*p*	OR (95%CI)
Age	0.05	0.01	4.05	< 0.001	1.05 (1.02–1.07)	0.04	0.01	3.03	0.002	1.04 (1.02–1.07)
Disturbance of consciousness										
No					1.00 (Reference)					1.00 (Reference)
Yes	1.56	0.34	4.62	< 0.001	4.74 (2.45–9.17)	1.63	0.41	3.94	< 0.001	5.10 (2.27–11.45)
Use glucocorticoids										
No					1.00 (Reference)					1.00 (Reference)
Yes	1.84	0.44	4.16	< 0.001	6.29 (2.64–14.95)	1.76	0.52	3.39	< 0.001	5.82 (2.10–16.12)
History of carbapenem use										
No					1.00 (Reference)					
Yes	0.92	0.37	2.48	0.013	2.51 (1.21–5.20)					
SOFA score	0.18	0.05	3.81	< 0.001	1.20 (1.09–1.31)	0.23	0.06	3.88	< 0.001	1.26 (1.12–1.42)
C-reactive protein	0.01	0.00	3.73	< 0.001	1.01 (1.01–1.02)	0.01	0.00	3.01	0.003	1.01 (1.01–1.02)
Albumin	−0.09	0.04	−2.05	0.041	0.92 (0.85–0.99)					

Subsequently, they were entered into a multivariate binary logistic regression model. This final model identified five independent risk factors for 30-day mortality (Table 4): older age [adjusted odds ratio (aOR) = 1.04, 95% CI: 1.02–1.07], impaired consciousness (aOR = 5.10, 95% CI: 2.27–11.45), a history of corticosteroid use (aOR = 5.82, 95% CI: 2.10–16.12), higher SOFA score (aOR = 1.26, 95% CI: 1.12–1.42), and elevated CRP level (aOR = 1.01, 95% CI: 1.01–1.02) (all *p* < 0.05).

### Development and validation of a nomogram for predicting 30-day mortality in ICU patients with CRAB-BSI

**Figure 3:**
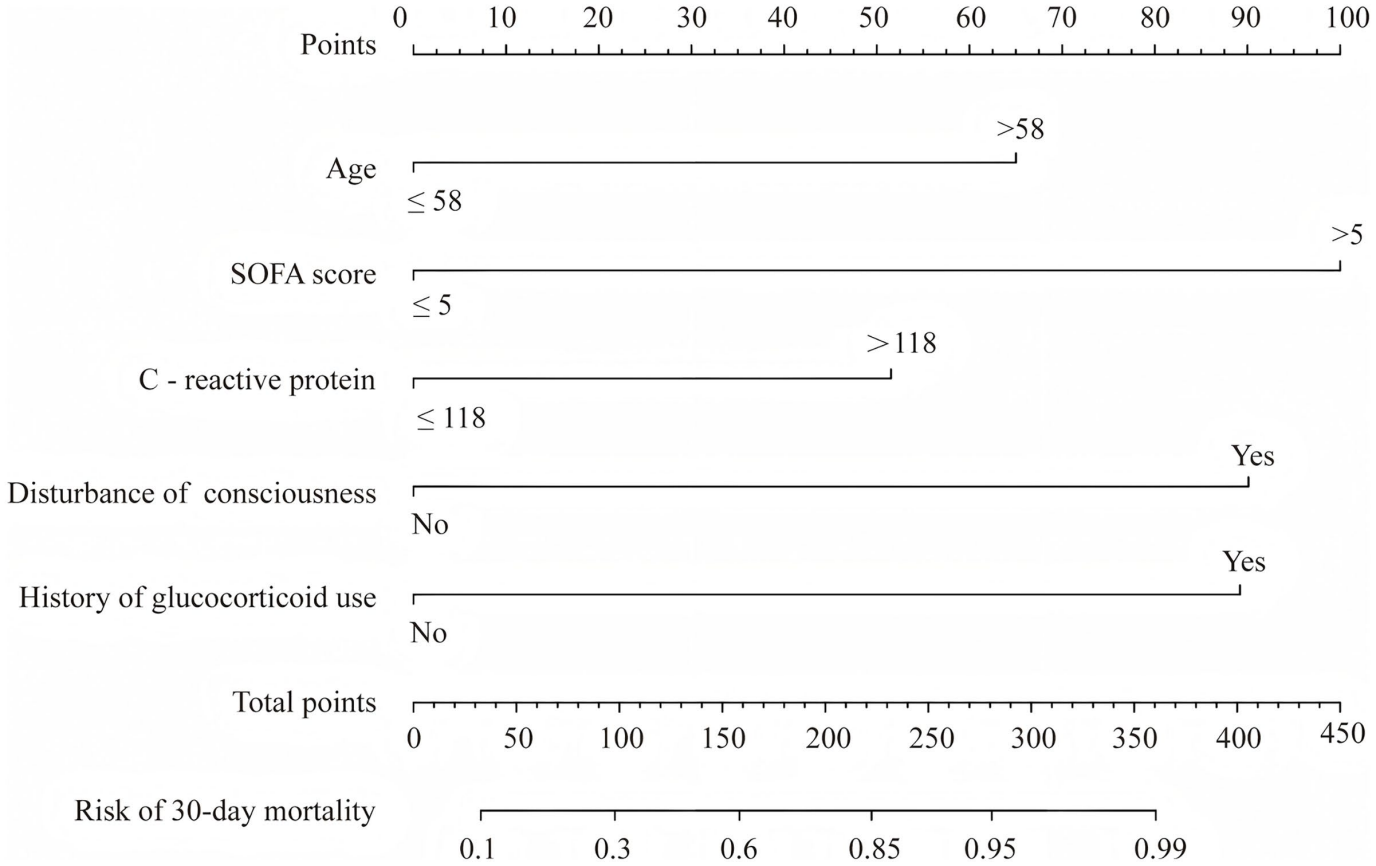
Nomogram for predicting the 30-day mortality of CRAB-BSI patients in ICU.

**Figure 4:**
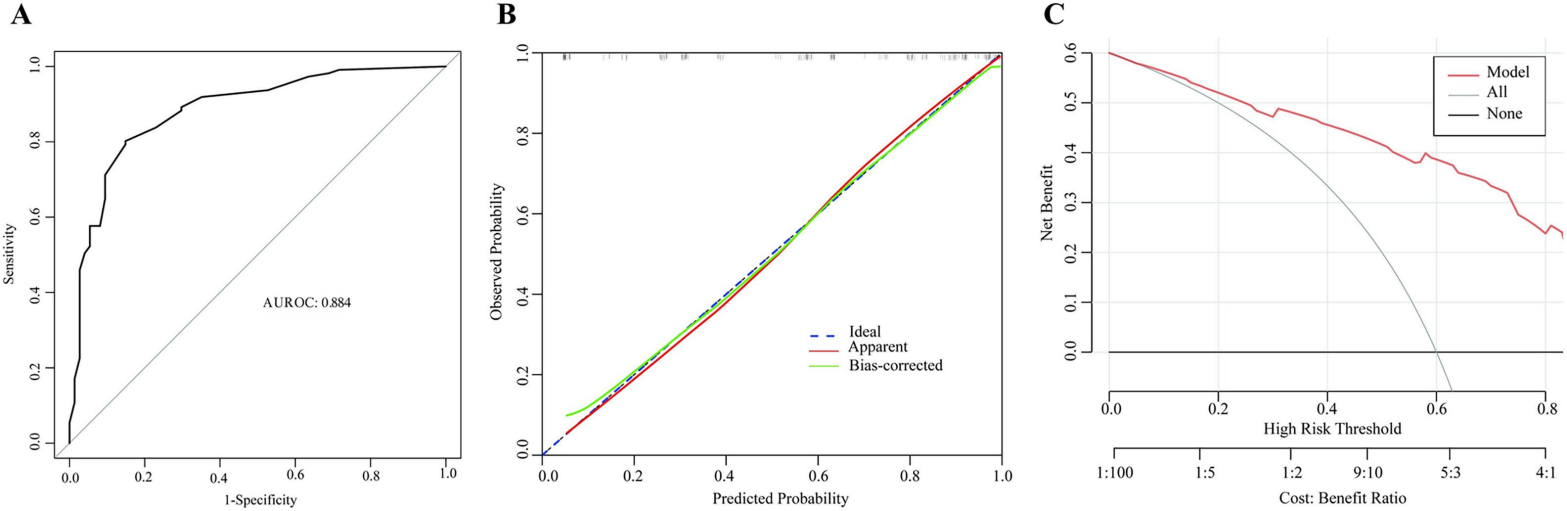
Performance evaluation of the nomogram. **(A)** Receiver operating characteristic (ROC) curve showing the model’s discrimination (AUROC = 0.863). **(B)** Calibration curve comparing predicted and observed mortality probabilities. **(C)** Decision curve analysis showing the net clinical benefit across threshold probabilities.

Based on the independent risk factors, a nomogram was constructed to predict 30-day mortality risk in ICU patients with CRAB-BSI (Figure 3). The optimal cutoff values for continuous variables, determined by receiver operating characteristic curve (ROC) analysis, were 58 years for age, 5.0 for SOFA score, and 118 mg/L for CRP. The nomogram achieved an AUROC of 0.863 (95% CI: 0.804–0.915) for predicting 30-day mortality, with a sensitivity of 85% and specificity of 76% (Figure 4A). Model calibration, assessed using the Hosmer-Lemeshow test, yielded a non-significant result (*χ*^2^ = 10.82, *p* = 0.212), indicating excellent concordance between the predicted and observed outcomes. The calibration curve further confirmed a satisfactory agreement between the predicted probabilities and actual observations (Figure 4B). Decision curve analysis revealed that the use of this nomogram provided a net clinical benefit across a wide threshold probability range of 0.10–0.92 (Figure 4C).

### Internal validation of the nomogram

**Figure 5:**
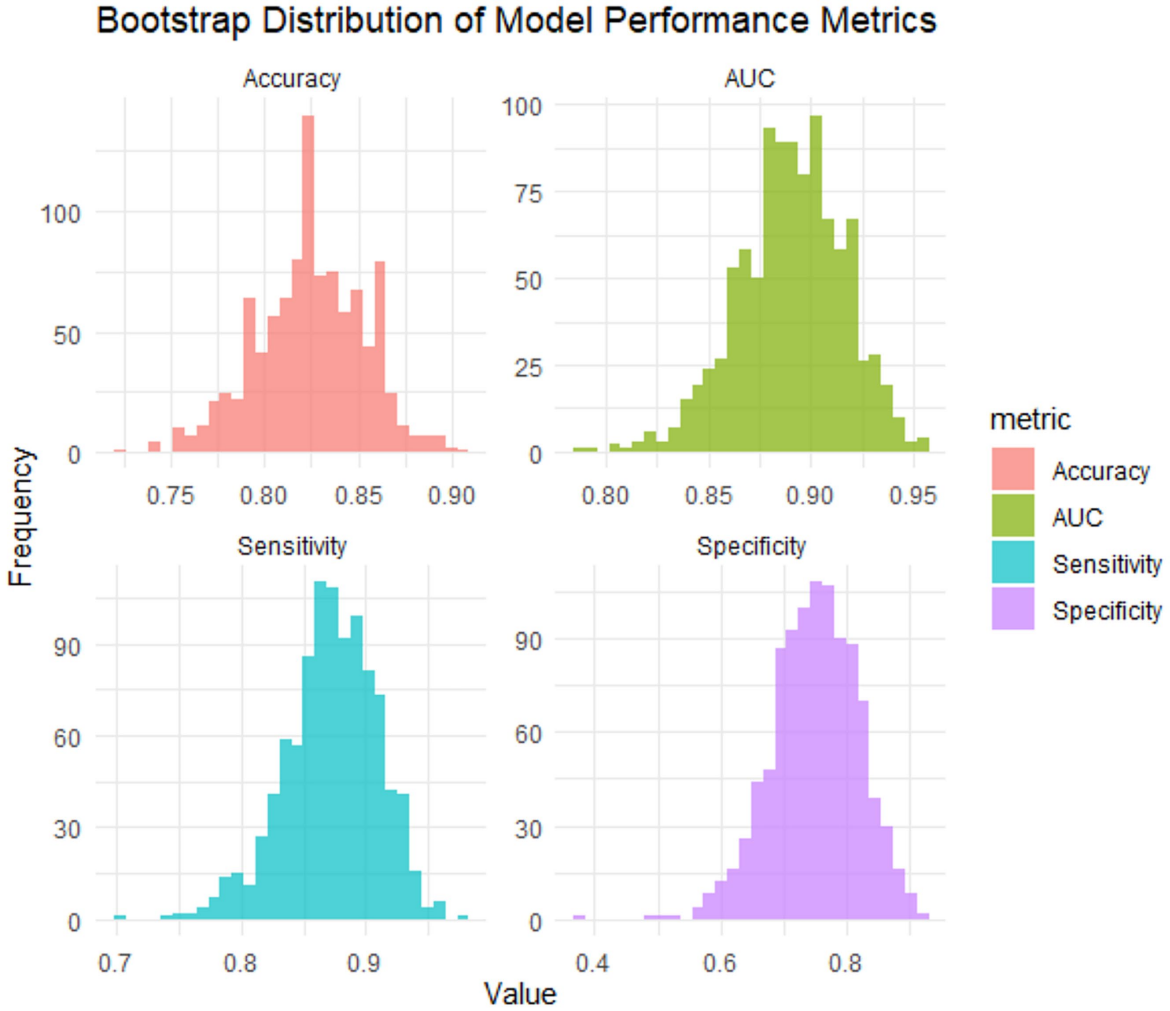
Performance distribution of the nomogram from bootstrap internal validation (1,000 replicates).

Internal validation was performed using bootstrap resampling with 1,000 replicates to assess model stability and correct for overfitting. The optimism-corrected performance metrics were as follows: AUROC = 0.890 (95% CI: 0.834–0.938), sensitivity = 0.873 (95% CI: 0.789–0.937), and specificity = 0.750 (95% CI: 0.608–0.875). These results confirmed the robustness and generalizability of the nomogram’s predictive performance (Figure 5).

## Discussion

This study substantiates that the prognosis of ICU-acquired CRAB-BSI is determined not merely by the antimicrobial resistance profile of the pathogen, but by a critical convergence of impaired host defense and the severity of the systemic inflammatory response. This paradigm shift is robustly supported by our microbiological findings, which revealed no significant difference in antimicrobial susceptibility profiles between survivors and non-survivors (Table 3). In an era of near-pan-resistance, where effective pathogen-targeted therapeutic options are severely constrained, patient survival hinges more decisively on the individual’s physiological reserve and the clinical response to the infective inflammatory insult. To operationalize this insight, we developed a nomogram prediction model that integrates key clinical markers reflecting these two dimensions—namely, indicators of host vulnerability and markers of inflammation/organ dysfunction—thereby translating a theoretical construct into a practical tool for rapid bedside assessment.

The independent risk factors identified in this study can be conceptualized within two interrelated dimensions: “baseline host vulnerability” and “the intensity of the infection-induced systemic inflammatory response.” The former defines the intrinsic risk reserve of a patient prior to encountering the infection, while the latter dynamically quantifies the pathophysiological burden imposed by the host’s defensive reaction post-infection.

Within the dimension of host vulnerability, advanced age (OR = 1.04) emerged as an independent risk factor, consistent with the established pathophysiology of immunosenescence and the decline in multi-organ physiological reserve associated with aging (38–40). Impaired consciousness was also significantly associated with mortality (OR = 5.10), a relationship underpinned by a dual mechanism. It serves both as a direct marker of severe systemic inflammation or septic encephalopathy (41) and as a clinical condition that elevates the risk of aspiration pneumonia and complicates airway management, potentially initiating a vicious cycle of clinical deterioration (42, 43). Furthermore, a history of glucocorticoid use—an iatrogenic vulnerability factor requiring consideration within its specific temporal context—demonstrated particularly strong predictive value.

A history of glucocorticoid use stood out as the strongest predictor (OR = 5.82), warranting nuanced clinical interpretation. This variable likely encapsulates two distinct yet complementary high-risk scenarios in critically ill patients: (1) a state of chronic immunosuppression due to maintenance therapy for an underlying condition, or (2) the emergent immunomodulatory treatment for an acute, severe inflammatory condition (e.g., septic shock, acute respiratory distress syndrome). In both scenarios, the common pathway to poor outcome is a compromised host defense at the time of bacteremic challenge—whether stemming from baseline immune dysfunction or from iatrogenic immunosuppression superimposed on critical illness. This finding strongly reinforces the paradigm that in CRAB-BSI, patient survival is often governed more by the functional reserve of the host than by the antimicrobial resistance profile of the pathogen (44–46).

The clinical relevance of these risk factors is further amplified when considered within the specific historical context of our study period. The interpretation of our findings, particularly the strong predictive value of prior corticosteroid use, must be carefully contextualized within the timeline of our study, which spanned the COVID-19 pandemic (2020–2023). This global health crisis constituted a defining event within our decade-long study period (2013–2023), profoundly altering ICU admission patterns, therapeutic protocols, and infection epidemiology worldwide (47, 48). While our inclusion criteria were based on a diagnosis of CRAB-BSI and did not stratify patients by COVID-19 status, our cohort objectively mirrors the evolution of the critically ill population before, during, and after this unique phase. Evidence-based guidelines during the pandemic led to the widespread use of glucocorticoids for severe pneumonia (49), inevitably increasing the proportion of ICU patients receiving such immunomodulatory therapy in the latter part of our study. Consequently, the variable “glucocorticoid use” likely captures the synergistic high-risk confluence of “extensive immunomodulatory therapy” and “a concentrated influx of critically ill hosts into the ICU” during the pandemic period. This highlights the paramount importance of iatrogenic immunosuppression as a marker of host vulnerability under specific temporal and therapeutic conditions. It further suggests that during future public health crises imposing similar strains on critical care systems, risk assessment for CRAB-BSI must remain highly vigilant for such acquired immunosuppressive factors.

Concurrently, our model incorporates direct measures of the severity of the systemic inflammatory response, offering a physiological perspective for early mortality risk assessment in CRAB-BSI. The Sequential Organ Failure Assessment (SOFA) score, a cornerstone for evaluating sepsis-related organ dysfunction (50, 51), was an independent predictor (OR = 1.26), underscoring that the degree of multi-organ failure is central to determining prognosis. The cutoff value (> 5) identified in our study provides an objective threshold for bedside risk recognition. C-reactive protein (CRP) level dynamically reflects the intensity of the systemic inflammatory response (41, 52, 53). In the context of near-pan-resistant CRAB infections and the increasingly complex clinical challenges exemplified by events like the COVID-19 pandemic, the prognostic value of parameters like SOFA and CRP—which reflect the host’s immediate physiological state—transcends that of purely microbiological data. This shift in focus is critical: it implies that within the therapeutic constraint of limited antimicrobial options, clinical decision-making can utilize these readily available parameters to prioritize and intensify organ support and inflammation modulation for patients sustaining the most severe physiological insult—representing a potentially pivotal pathway toward improving outcomes.

As a major manifestation of CRAB infection, bloodstream infection (BSI) and ventilator-associated pneumonia (VAP) represent two common and life-threatening infections in the ICU. Although they differ in primary site and initial clinical presentation, they share significant overlaps in underlying risk factors, pathophysiological mechanisms, and therapeutic challenges. Both are frequently observed in mechanically ventilated, multi-site colonized, and immunocompromised hosts (54, 55) and are associated with alarmingly high mortality rates (56, 57). Notably, the incidence of both CRAB-BSI and CRAB-VAP increased in ICUs during the COVID-19 pandemic (30), indicating they are jointly influenced by host immune status and healthcare environmental pressures. Therefore, in clinical management, risk assessment and early intervention strategies for these severe CRAB infections should be conceptually aligned, with a shared emphasis on monitoring host vulnerability and the systemic inflammatory response. Although developed and validated specifically for BSI, the nomogram presented in this study—which integrates age, consciousness status, corticosteroid use, SOFA score, and CRP—is fundamentally built upon these shared pathophysiological pillars. Thus, it may also provide a valuable conceptual framework and a practical reference for initial risk stratification in patients with other severe, systemic CRAB infections, such as VAP, warranting future investigation in those specific cohorts.

Actually, the developed nomogram effectively translates the identified risk paradigm into a pragmatic bedside tool, demonstrating strong predictive performance that holds immediate potential for clinical application. Its utility lies in leveraging parameters such as age, consciousness status, corticosteroid use, SOFA score, and CRP, which are routinely and rapidly available in the ICU, often within the first hours of admission or at the time of CRAB-BSI suspicion. This facilitates integration into clinical workflows at several junctures. First, for risk stratification and triage, a high-risk score can flag patients for intensified monitoring (such as invasive hemodynamic monitoring) and prompt consideration for early, aggressive organ support in a higher-acuity setting. Second, to inform the initial therapeutic strategy, where the quantified mortality risk can guide empirical antibiotic choices while awaiting definitive susceptibility results, favoring combination regimens or newer agents for patients at the highest risk of adverse outcomes. Third, to enhance prognostic communication, provides an objective, evidence-based estimate to structure discussions with patients’ families regarding the anticipated clinical course and the rationale for invasive interventions. Embedding this algorithm as a clinical decision support module within the electronic health record could automate calculation and provide real-time alerts, thereby standardizing risk assessment and promoting timely, guideline-concordant care.

The nomogram exhibited strong predictive performance within the study cohort, as evidenced by an AUROC = 0.863, excellent calibration, and positive net benefit on decision curve analysis. Certain study limitations should be considered. The retrospective, single-center design of this study inherently carries risks of selection bias and may limit the generalizability of our findings. Specifically, caution is warranted when interpreting certain risk factors. For instance, the strong association with corticosteroid use likely reflects the unique patient mix and prevailing treatment practices at our institution, particularly the surge of COVID-19 pneumonia patients receiving immunomodulatory therapy during the pandemic. The operational definition of ‘glucocorticoid use’ warrants careful consideration. In this retrospective study, it was designed as a pragmatic, binary variable capturing any documented systemic use prior to bacteremia. While this ensures sensitivity in identifying patients exposed to iatrogenic immunosuppression, it inherently encompasses substantial heterogeneity. We lacked granular data on specific agents, cumulative doses, treatment durations, and precise clinical indications (e.g., chronic maintenance therapy versus short-course pulse therapy for acute inflammatory conditions). Consequently, our variable serves best as a robust composite marker of ‘significant immunosuppressive exposure’ rather than a precise pharmacologic measure. The strong association observed (OR = 5.82) underscores the profound risk conferred by such exposure in the context of CRAB-BSI. However, our study design cannot elucidate dose–response relationships or define a minimum risk threshold. Future prospective studies should aim to collect detailed corticosteroid exposure data to refine this risk factor, potentially distinguishing between different patterns of use (e.g., high-dose acute vs. low-dose chronic) for more personalized risk assessment. A more fundamental limitation lies in the model’s scope. By design, it incorporates only baseline clinical variables and excludes detailed data on subsequent antimicrobial therapy (e.g., appropriateness, timing, specific agents) and source control interventions. As underscored by recent research (17, 58), these treatment factors are critical, modifiable determinants of outcome in drug-resistant infections. Consequently, our nomogram is best understood and applied as a tool for initial risk stratification at the time of CRAB-BSI diagnosis, providing a pre-therapeutic risk estimate. It does not account for the impact of subsequent clinical decisions. To translate this tool into broader practice, external validation is imperative. Future studies must assess its performance across diverse geographical and epidemiological settings, particularly in centers with different CRAB prevalence rates and patient demographics, to confirm its transportability before widespread clinical adoption.

## Conclusion

In conclusion, this study identifies age, impaired consciousness, prior corticosteroid use, higher SOFA score, and elevated CRP level as independent risk factors for 30-day mortality in critically ill patients with CRAB-BSI. By integrating these five readily available clinical parameters, we developed and internally validated a nomogram that accurately predicts mortality risk. This pragmatic tool may aid in the early bedside risk stratification of patients with this life-threatening infection, helping to guide clinical decision-making. External validation is warranted before widespread clinical application.

## Data Availability

The raw data supporting the conclusions of this article will be made available by the authors, without undue reservation.
